# A Case of Morphine Poisoning Treated with Permanganate of Potash

**Published:** 1894-09

**Authors:** J. I. Darby

**Affiliations:** Americus, Ga.


### A CASE OF MORPHINE POISONING TREATED WITH PERMANGANATE OF POTASH.

BY J. I. DARBY, M.D.,

AMERICUS, GA.

Having seen some articles in the medical journals of late in regard to the treatment of morphine poisoning with permanganate of potash, and having recently had some experience with it in an extreme case, I feel it my duty to report the following case:

On the morning of July 11th I was called by telephone at 5 : 30 o'clock to attend J. H., of this city, a mechanic, aged fifty-four years, who had six hours previously swallowed twelve grains of sulphate of morphine in solution. His wife stated that about one o'clock that night he awoke her and said that he had not slept any and that he had just taken some morphine; but as he had a few times previously taken a quarter grain pellet for insomnia, nothing serious was thought of it, and he soon fell asleep. At about five o'clock next morning the attention of the family was called to his heavy, stertorous breathing and profound sleep. Attempts to awake him soon revealed the fact that he was deeply under the narcotic influence of the morphia, with limbs rigid, skin cyanosed and pupils contracted. I reached him about thirty minutes later, and found him in the condition above described, but by vigorously shaking, rubbing, dashing cold water on him and screaming loudly in his ears, succeeded in getting him to swallow about three grains of permanganate of potash dissolved in a small quantity of water, and also a cupful of strong, hot coffee. I gave him hypodermically onefiftieth grain of sulphate atropia, but not knowing how much morphine he had taken, I went to the nearest telephone and learned from the druggist whose name was on the empty envelope found on his table, marked morphine, that he had, on his way home from work the previous evening, bought twelve grains of sulphate of morphine in powder. His condition and the empty envelope made it very suspicious that he had taken the entire twelve grains, and subsequent developments confirmed this suspicion. Nowithstanding the permanganate, the coffee and the hypodermic of atropine, and all possible efforts to keep him awake, he soon lapsed into a profound sleep from which it was impossible to arouse him. His respirations very soon went down to four per minute, and they were superficial and stertorous in the extreme, with intervals occasionally so long between breaths that it appeared as if he would never breathe again. The heart's action was full and strong, numbering about ninety beats per minute, while the veins were full and the capillary system congested with dark venous blood. Artificial respiration was promptly instituted and kept up until a faradic battery was procured and one electrode placed immediately over the phrenic nerve in the neck and the other over the epigastrium, and a strong current of electricity turned on, which, in a short time, increased the number of respirations to eight or ten per minute, but they were very shallow and imperfect.

There was very little response when the electricity was first applied, even the muscles scarcely contracting under a very strong current. Two-grain doses of caffeine were given hypodermically twice during the forenoon, and his urine drawn with catheter. The caffeine produced no decided effect, unless it was to stimulate and slightly quicken the respirations, which immediately failed whenever the electricity was for a moment withheld. He continued in this condition with but little change, except when the electricity was withheld, until about two o'clock in the afternoon, when his heart suddenly grew weak, and his respirations would have stopped entirely but for the prompt resort to artificial respiration and a hypodermic of one-thirtieth grain nitrate of strychnine. His condition at this time had become so serious that no one had any hope of his recovery, but as a last resort, I resolved, against my better judgment, to try the permanganate hypodermically, and accordingly dissolved two grains of it in a small quantity of warm water and gave him two ordinary hypodermic syringefuls, one in each arm, every half hour until the two grains were given. In the meantime, the electricity was kept up for an hour or two longer, when it became apparent that his breathing was more frequent and decidedly deeper, which in a short time began to improve the appearance of his complexion, which took on a more natural appearance. At six o'clock P. M. his pupils were very much contracted, and he was given one-fiftieth grain sulphate atropia, and I left him in care of a competent nurse, and went home for an hour and returned to find him still breathing better, respirations numbering fourteen per minute and his skin much less cyanosed. The urine was again drawn off and another grain of the permanganate given, which very soon seemed to further benefit his respirations, and at nine o'clock P. M. he opened his eyes, recognized those around him and drank a cupful of hot coffee, from which time his condition steadily improved, and in due time he made a satisfactory recovery. I know that it is contrary to the generally accepted opinion of medical men that permanganate of potash acts in these cases otherwise than by rendering the morphine inert in the stomach before it is absorbed into the circulation, but in this case I am persuaded it did more. I believe it saved this man's life by its effect upon the poison in his blood, as the time had been too long after taking it before the remedy was given for it to have done its work in the stomach, and I report the case to induce others to try it in similar cases.

I would always put it in the patient's stomach while he was able to swallow, and give it hypodermically when too profoundly comatosed to take it by the mouth. I think it well in these cases to use other recognized remedies, such as caffeine, atropine, strychnia and electricity, remembering that permanganate can be given in poisonous doses, and thereby do more harm than good. I think this remedy well worth trying hypodermically in any extreme case of morphine poisoning.

